# Erectile dysfunction and lower urinary tract symptoms in men with multiple sclerosis

**DOI:** 10.1055/s-0046-1829050

**Published:** 2026-10-02

**Authors:** Luciano Gonçalves do Nascimento Júnior, Alex Tiburtino Meira, Felipe Nunes de Araújo, Bianca Etelvina Santos de Oliveira

**Affiliations:** 1 Hospital Metropolitano Dom José Maria Pires, Neurology Service, João Pessoa PB, Brazil.; 2 Universidade Federal da Paraíba, Department of Internal Medicine, João Pessoa PB, Brazil.; 3 Centro de Referência em Esclerose Múltipla do Estado da Paraíba, João Pessoa PB, Brazil.

**Keywords:** Multiple Sclerosis, Erectile Dysfunction, Lower Urinary Tract Symptoms, Disability Evaluation, Penile Erection

## Abstract

**Background:**

Multiple sclerosis (MS) is a chronic demyelinating disease of the central nervous system commonly associated with lower urinary tract symptoms (LUTS) and erectile dysfunction (ED); however, these conditions remain underinvestigated in Brazilian populations.

**Objective:**

To evaluate the prevalence of ED and LUTS, to analyze the correlations involving the 5-item International Index of Erectile Function (IIEF-5), the International Prostate Symptom Score (IPSS), the Expanded Disability Status Scale (EDSS), age, and disease duration, and to identify the subscale items most strongly associated with neurological disability in men with MS.

**Methods:**

We conducted a cross-sectional study enrolling 56 men with MS diagnosed through the McDonald criteria at the Reference Center for MS of the state of Paraíba, Brazil. Erectile dysfunction was assessed using the IIEF-5, and LUTS, using the IPSS. Spearman correlations with Bonferroni correction, univariate linear regression, one-way analysis of variance (ANOVA), and Tukey's Honestly-Significant Difference (HSD) post-hoc tests were performed.

**Results:**

The mean age of the sample was 39.9 ± 12.3 years, the median EDSS score, 2.75, and the median disease duration, 7 years. The prevalence of ED was of 50%, and that of LUTS, 96.4%. Strong correlations were found between the scores on the EDSS and the IIEF-5, the EDSS and the IPSS, and the IIEF-5 and the IPSS. Through the analysis, we identified erection confidence and urinary intermittency as the subscale items most strongly associated with the EDSS score. One-way ANOVA revealed significant differences across MS phenotypes for the IIEF-5, IPSS, and EDSS, with Tukey HSD confirming relapsing-remitting MS versus secondary progressive MS as the primary source of divergence.

**Conclusion:**

Erectile dysfunction and LUTS were highly-prevalent in men with MS in the cohort of the present study. Disability level was the strongest correlate of both dysfunctions, supporting integrated urological-neurological screening in MS care.

## INTRODUCTION

Multiple sclerosis (MS) is a chronic autoimmune demyelinating and inflammatory disease of the central nervous system (CNS), characterized by multifocal and temporally-disseminated lesions. 1 In 2020, the global prevalence of MS was estimated 2 at 35.9 cases per 100 thousand individuals, corresponding to approximately 2.8 million people worldwide. Multiple sclerosis predominantly affects young adults, with significant implications for sexual and urological function. 3

Lower urinary tract symptoms (LUTS) are among the most common autonomic manifestations in MS, with prevalence rates ranging from 73 to 97.6% across different populations. 4 5 6 Urinary symptoms typically appear 6 to 8 years after MS diagnosis, and they tend to worsen with increasing disability. 7 Storage symptoms, particularly urgency and nocturia, are the most frequently-reported urinary complaints. 4 5

Erectile dysfunction (ED) is the most common sexual complaint among men with MS. Meta-analytic data 8 indicate a pooled ED prevalence of 49% (95%CI: 42–56%) across 2,760 men, with a relative risk of 3.17 compared with controls. When assessed with the full version of the International Index of Erectile Function (IIEF), prevalence estimates rise to approximately 63%. 8 The pathophysiology involves disruption of spinal and supraspinal autonomic pathways that regulate micturition and penile erection, resulting in the frequent co-occurrence of LUTS and ED. 9 10

Despite the high prevalence of these conditions, urological and sexual issues remain underassessed in routine MS care, particularly in developing countries. 11 Brazilian data on ED and LUTS in men with MS are scarce, limited to a single study from the state of São Paulo, Southeastern Brazil. 6 No previous study has evaluated these outcomes in the Northeastern region of Brazil. The present study aimed to assess the prevalence of ED and LUTS and to investigate the correlations involving the scores on the 5-item IIEF (IIEF-5), the International Prostate Symptom Score (IPSS), the Expanded Disability Status Scale (EDSS), age, and disease duration in men with MS.

## METHODS

### Study design and population

The current was an observational, cross-sectional, analytical study conducted at the MS Reference Center of the State of Paraíba (Centro de Referência em Esclerose Múltipla do Estado da Paraíba, CREMPB, in Portuguese), located at Fundação Centro Integrado de Apoio à Pessoa com Deficiência (FUNAD), in the capital city of João Pessoa, Paraíba, from August to December 2022. The study was approved by the Research Ethics Committee of Centro Universitário de João Pessoa (UNIPÊ), under certificate of ethical appreciation number 59231422.0.0000.5176. Written informed consent was obtained from all participants.

The study population comprised male MS patients diagnosed according to the McDonald criteria. The inclusion criteria were age over 18 years, active sexual life with sexual intercourse in the preceding 6 months, complete remission of MS relapses for at least 6 months, and an EDSS score below 8.0. The exclusion criteria were prostatic volume greater than 30 g (measured by transabdominal ultrasonography), urinary retention requiring indwelling catheter, history of pelvic or prostate surgery, prior pelvic radiotherapy, bladder stones, urethral stricture, and continuous use of 5-α-reductase inhibitors. Of 98 eligible male patients, 56 met all criteria and were included.

### Clinical and score assessments

Erectile function was assessed using the validated Brazilian Portuguese version of the IIEF-5 (score: 5–25; ≤ 21 indicates ED). 12 13 The severity of ED was classified as: mild (17–21), mild-to-moderate (12–16), moderate (8–11), or severe (5–7). 13 The LUTS were evaluated with the validated Brazilian Portuguese version of the IPSS (scores: 0–35), which are categorized as none (0), mild (1–7), moderate (8–19), or severe (20–35). 14 15 Neurological disability was assessed using the EDSS. 16 Demographic and clinical variables included age, race, marital status, disease duration (from symptom onset) and MS phenotypes: relapsing-remitting MS (RRMS); secondary progressive MS (SPMS); progressive relapsing MS (PRMS); and primary progressive MS (PPMS).

### Statistical analysis

The normality of data distribution was assessed using the Shapiro-Wilk test. The continuous variables were expressed as mean ± standard deviation or median (interquartile range; IQR) values, according to normality. The categorical variables were expressed as absolute and relative frequencies. Spearman rank correlation coefficients (ρ) were used to evaluate bivariate correlations between the scores on the EDSS, IIEF-5, and IPSS, as well as age, and disease duration. Bonferroni correction was applied to account for 10 pairwise correlation comparisons (adjusted significance threshold α = 0.005). For phenotype comparisons, PPMS and PRMS were combined into a single progressive primary group (PP). One-way analysis of variance (ANOVA) was used to compare the IIEF-5, IPSS, and EDSS scores across phenotypes, followed by Tukey's Honestly-Significant Difference (HSD) post-hoc test for pairwise comparisons. Individual item-level scores of the IIEF-5 and IPSS were examined as secondary predictors of the EDSS score in the univariate Spearman correlation and linear regression analyses; these item-level analyses were exploratory and hypothesis-generating rather than prespecified, and effect-size interpretations should therefore be considered preliminary. Univariate linear regression coefficients (β) were reported with standard errors (SEs), 95%CIs, and coefficients of determination (R ^2^ ); Wilson 95%CIs were calculated for proportions across EDSS bands. Statistical significance was set at *p* < 0.05 (uncorrected) and *p* < 0.005 (Bonferroni-adjusted, for correlation analyses). All analyses were performed using the IBM SPSS Statistics for Windows (IBM Corp.) software, version 29.0.2, and the Python 3.11 software (free and open source).

## RESULTS

A total of 56 men with MS were included. The demographic and clinical characteristics are presented in Table 1 . The mean age was 39.9 ± 12.3 (range: 18–69) years. Most participants were married (60.7%), of mixed race (51.8%), and had RRMS (75.0%). The median EDSS score was 2.75 (IQR: 2.0–4.5), and the median disease duration was 7 (IQR: 4.75–15.0) years.

**Table 1:** Demographic, clinical, and outcome characteristics of men with multiple sclerosis ( *n* = 56)

Variable	Value
Demographics	
Mean age (years)	39.9 ± 12.3
Race: n (%)	
White	24 (42.9)
Mixed	29 (51.8)
Black	3 (5.4)
Marital status: n (%)	
Single	21 (37.5)
Married	34 (60.7)
Divorced	1 (1.8)
Clinical characteristics	
MS phenotype: n (%)	
RRMS	42 (75.0)
SPMS	10 (17.9)
PPMS	3 (5.4)
PRMS	1 (1.8)
Disease duration: (years): median (IQR) ^†^	7.0 (4.75–15.0)
EDSS score: median (IQR) ^†^	2.75 (2.0–4.5)
Outcome measures	
IIEF-5 score: median (IQR) ^†^	21.5 (14.75–24.0)
ED prevalence (IIEF-5 ≤ 21): n (%)	28 (50.0)
Mild (17–21)	10 (17.9)
Mild-to-moderate (12–16)	9 (16.1)
Moderate (8–11)	4 (7.1)
Severe (5–7)	5 (8.9)
IPSS score: median (IQR) ^†^	12.0 (7.0–20.25)
LUTS prevalence (IPSS ≥ 1): n (%)	54 (96.4)
Mild (1–7)	18 (32.1)
Moderate (8–19)	19 (33.9)
Severe (20–35)	17 (30.4)

Abbreviations: ED, erectile dysfunction; EDSS, Expanded Disability Status Scale; IIEF-5, 5-Item International Index of Erectile Function; IPSS, International Prostate Symptom Score; IQR, interquartile range; LUTS, lower urinary tract symptoms; MS, multiple sclerosis; PPMS, primary progressive MS; PRMS, progressive relapsing MS; RRMS, relapsing-remitting MS; SPMS, secondary progressive MS.

Notes: The continuous variables are expressed as mean ± standard deviation (normally-distributed) or median (IQR) values (non-normally-distributed) per the Shapiro–Wilk test. ^†^ Non-normally-distributed variable.

The prevalence of ED (IIEF-5 ≤ 21) was of 50% ( *n* = 28). Among those with ED, the distribution was: mild in 17.9% ( *n* = 10), mild-to-moderate in 16.1% ( *n* = 9), moderate in 7.1% ( *n* = 4), and severe in 8.9% ( *n* = 5). The median IIEF-5 score was 21.5 (IQR: 14.75–24.0) ( Table 1 ). The IIEF-5 domain with the lowest mean score was erection confidence (3.27 ± 1.20), while the highest was maintenance to completion (4.02 ± 1.36).

Lower urinary tract symptoms were present in 96.4% ( *n* = 54) of the participants. The severity distribution was mild in 32.1% ( *n* = 18), moderate in 33.9% ( *n* = 19), and severe in 30.4% ( *n* = 17). Only 2 patients (3.6%) reported no urinary symptoms ( Table 1 ). The median IPSS score was 12.0 (IQR: 7.0–20.25). Nocturia had the highest mean score among IPSS items (2.7 ± 2.0), while straining had the lowest (1.3 ± 1.6).

When stratified by disability level, ED and LUTS severity showed a marked gradient across EDSS bands ( Figure 1 ). Among patients with EDSS 0–2.5 ( *n* = 28), 82% had no ED, and only 18% presented mild dysfunction; no patient in this band had moderate or severe ED. In contrast, among those with EDSS 3.0–5.5 ( *n* = 16), ED prevalence rose to 75%, with 38% classified as mild-to-moderate and 12%, as severe. Among those with EDSS ≥ 6.0 ( *n* = 12), 92% had ED, with the majority (58%) presenting moderate-to-severe forms. The Wilson 95%CIs for ED prevalence were: 17.9% (7.9–35.6%) for EDSS 0–2.5; 75.0% (50.5–89.8%) for EDSS 3.0–5.5; and 91.7% (64.6–98.5%) for EDSS ≥ 6.0. A parallel gradient was observed for LUTS: while 50% of the patients with EDSS 0–2.5 had only mild symptoms, 100% of those with EDSS ≥ 6.0 had severe LUTS (IPSS ≥ 20). No patient with EDSS ≥ 6.0 had mild or absent urinary symptoms. The corresponding Wilson 95%CIs for moderate-to-severe LUTS (IPSS ≥ 8) were 42.9% (26.5–60.9%) for EDSS 0–2.5; 75.0% (50.5–89.8%) for EDSS 3.0–5.5; and 100% (75.8–100%) for EDSS ≥ 6.0, reproducing the same threshold pattern observed for ED.

**Figure 1:**
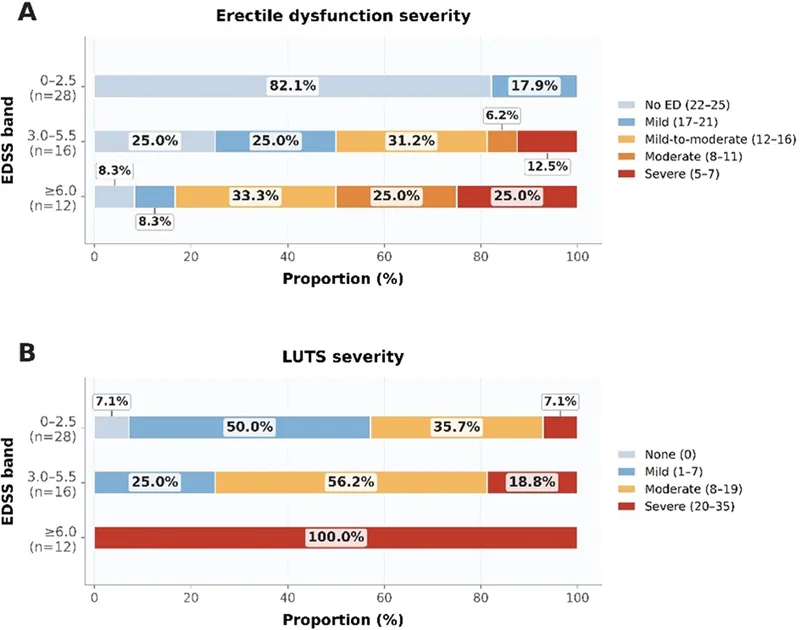
Severity distribution of erectile dysfunction (ED) ( **A** ) and lower urinary tract symptoms (LUTS) ( **B** ) stratified by Expanded Disability Status Scale (EDSS) band in men with multiple sclerosis (MS).

The co-occurrence analysis revealed substantial overlap between ED and moderate-to-severe LUTS. Of 56 patients, 25 (44.6%) presented both dysfunctions simultaneously, while only 3 (5.4%) had isolated ED without significant urinary symptoms. Conversely, 11 patients (19.6%) had moderate-to-severe LUTS without ED, and 17 (30.4%) had neither dysfunction. Among men with ED, 89.3% (25/28) also had moderate-to-severe LUTS. Among those with moderate–severe LUTS, 69.4% (25/36) also had ED.

One-way ANOVA revealed significant differences across MS phenotypes for all three outcomes ( Table 2 ). The RRMS patients had significantly better mean IIEF-5 (20.67 ± 4.08) and IPSS (11.43 ± 7.62) scores compared with the SPMS patients (IIEF-5: 11.60 ± 7.85; IPSS: 26.30 ± 9.13). Tukey's HSD post-hoc testing confirmed RRMS versus SPMS as the primary source of significant difference for all variables.

**Table 2:** Comparison of IIEF-5, IPSS, and EDSS scores across MS phenotypes: one-way ANOVA with Tukey HSD post-hoc pairwise comparisons

	RRMS	SPMS	PP	*F* (2.53)	*p*	Tukey HSD post-hoc		
Variable	*(n = 42)*	*(n = 10)*	*(n = 4)*			*RRMS* *×* *SPMS*	*RRMS* *×* *PP*	*SPMS* *×* *PP*
IIEF-5	20.67 ± 4.08	11.60 ± 7.85	14.75 ± 6.07	14.34	< 0.001	< 0.001	0.073	0.543
IPSS	11.43 ± 7.62	26.30 ± 9.13	15.75 ± 13.63	12.89	< 0.001	< 0.001	0.581	0.092
EDSS	2.81 ± 1.40	5.95 ± 1.01	4.25 ± 1.73	22.12	< 0.001	< 0.001	0.118	0.098

Abbreviations: ANOVA, analysis of variance; EDSS, Expanded Disability Status Scale; 'HSD, honestly-significant difference; IIEF-5, 5-Item International Index of Erectile Function; IPSS, International Prostate Symptom Score; MS, multiple sclerosis; RRMS, relapsing-remitting MS; SPMS, secondary progressive MS; PP, progressive MS (PPMS and PRMS combined).

Notes: Values expressed as mean ± standard deviation. Bold *p* -values indicate statistical significance ( *p* < 0.05).

Spearman correlations demonstrated strong associations ( Figure 2 ) between the EDSS and IIEF-5 (ρ = -0.764; *p* < 0.001), EDSS and IPSS (ρ = 0.637; *p* < 0.001), and IIEF-5 and IPSS (ρ = −0.673; *p* < 0.001) scores. Age correlated with the IIEF-5 (ρ = − 0.341; *p* = 0.010) and IPSS (ρ = 0.429; *p* = 0.001) scores. Disease duration correlated with the IPSS (ρ = 0.385; *p* = 0.003) score, but not with the IIEF-5 (ρ = −0.185; *p* = 0.171) score. After Bonferroni correction for 10 pairwise comparisons (α = 0.005), the primary correlations remained highly significant (EDSS–IIEF-5, p _Bonf_ < 0.001; EDSS–IPSS, p _Bonf_ < 0.001; IIEF-5–IPSS, p _Bonf_ < 0.001; IPSS–age, p _Bonf_ = 0.010; and IPSS–disease duration, p _Bonf_ = 0.034). The associations between EDSS and age (p _Bonf_ = 0.032), IIEF-5 and age (p _Bonf_ = 0.102), and EDSS and disease duration (p _Bonf_ = 0.309) were weaker, secondary contributors to urogenital outcomes.

**Figure 2:**
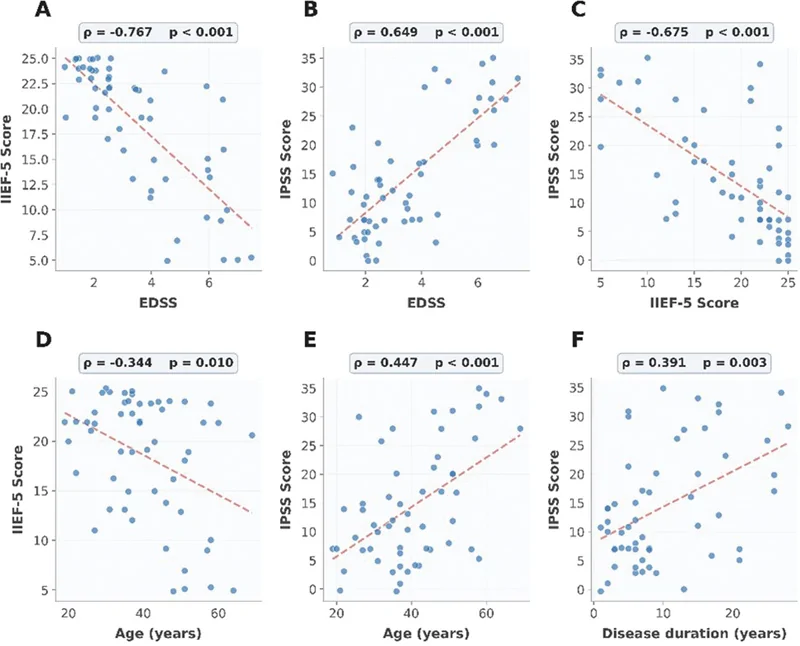
Scatter plots showing Spearman correlations between clinical variables. ( **A** ) EDSS versus 5-item International Index of Erectile Function (IIEF-5); ( **B** ) EDSS versus International Prostate Symptom Score (IPSS); ( **C** ) IIEF-5 versus IPSS; ( **D** ) age versus IIEF-5; ( **E** ) age versus IPSS; and ( **F** ) disease duration ersuvs IPSS.

The item-level analysis ( Tables 3 4 ) revealed that all IIEF-5 and IPSS subscores correlated significantly with the EDSS ( Figure 3 ) score. Within the ED domain, confidence in achieving an erection yielded the strongest association (ρ = −0.758), followed by postpenetration erection maintenance (ρ = −0.716) and erection firmness (ρ = −0.683); in the univariate regression, confidence in achieving an erection alone accounted for 59.1% of the variance in EDSS scores (R ^2^ = 0.591; β = −1.166; SE = 0.132; 95%CI: −1.431 to −0.901). Within the LUTS domain, urinary intermittency was the single most informative subitem (ρ = 0.610; β = 0.599; SE = 0.092; 95%CI: 0.414–0.784; R ^2^ = 0.438), ahead of urgency (ρ = 0.556) and incomplete bladder emptying (ρ = 0.539). Straining and urinary frequency showed the weakest (though still significant) associations with disability (ρ = 0.396 and 0.423 respectively). Complete regression statistics for all IIEF-5 and IPSS items and total scores are provided in **Supplementary Material Table S1** (available at https://www.arquivosdeneuropsiquiatria.org/wp-content/uploads/2026/06/ANP-2026.0146-Supplementary-Material.pdf ).

**Figure 3:**
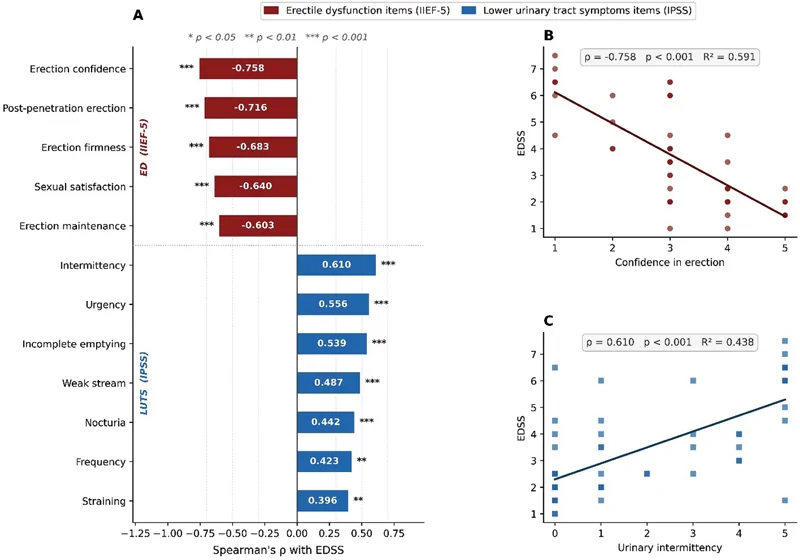
Association of ED and LUTS subscores with neurological disability (EDSS) in men with MS. ( **A** ) Spearman's correlation coefficients (ρ) between each IIEF-5 and IPSS subitem and the EDSS, ordered by effect size within domain; only items that reached statistical significance were presented. ( **B** ) Scatter plot illustrating the strongest ED subitem predictor: confidence in achieving erection. ( **C** ) Scatter plot illustrating the strongest LUTS subitem predictor: urinary intermittency. Regression lines derived from ordinary least squares.

**Table 3:** Scores on the IIEF-5 items by MS phenotype

Item	Median (IQR)	Prevalence n/N (%)	RRMS ( *n* = 42)	SPMS ( *n* = 10)	PP ( *n* = 4)
Erection confidence	3.0 (3.0–4.0)	47/56 (84%)	4.0 (3.0–4.0)	1.5 (1.0–2.8)	2.5 (1.8–3.5)
Erection firmness	4.0 (2.0–5.0)	31/56 (55%)	4.5 (4.0–5.0)	1.0 (1.0–4.2)	2.0 (2.0–2.8)
Postpenetrationerection	4.0 (3.0–5.0)	34/56 (61%)	4.0 (4.0–5.0)	1.0 (1.0–4.0)	2.5 (2.0–3.5)
Sexual satisfaction	4.0 (3.0–5.0)	32/56 (57%)	4.5 (4.0–5.0)	2.0 (1.0–3.8)	2.5 (2.0–3.5)
Erection maintenance	5.0 (4.0–5.0)	27/56 (48%)	5.0 (4.0–5.0)	1.0 (1.0–5.0)	3.5 (2.5–4.2)

Abbreviations: IQR, interquartile range; IIEF-5, 5-Item International Index of Erectile Function; MS, multiple sclerosis; RRMS, relapsing-remitting MS; SPMS, secondary progressive MS; PP, progressive MS (PPMS and PRMS combined).

Note: IIEF-5 items: score 1–5; impaired if score < 5.

**Table 4:** Scores on the IPSS items by MS phenotypes

Item	Median (IQR)	Prevalence: n/N (%)	RRMS ( *n* = 42)	SPMS ( *n* = 10)	PP ( *n* = 4)
Intermittency	1.0 (0.0–4.0)	37/56 (66%)	1.0 (0.0–2.8)	5.0 (5.0–5.0)	2.0 (0.8–3.5)
Urgency	1.0 (0.0–5.0)	33/56 (59%)	1.0 (0.0–3.0)	5.0 (5.0–5.0)	0.5 (0.0–2.0)
Incomplete emptying	2.0 (0.8–4.0)	42/56 (75%)	1.5 (0.0–3.0)	4.0 (2.0–5.0)	1.5 (0.8–2.8)
Weak stream	1.0 (0.0–3.0)	40/56 (71%)	1.0 (0.0–2.0)	4.0 (3.2–5.0)	1.0 (0.8–2.0)
Nocturia	2.0 (1.0–5.0)	50/56 (89%)	1.0 (1.0–4.0)	5.0 (5.0–5.0)	5.0 (4.0–5.0)
Frequency	2.0 (1.0–4.0)	51/56 (91%)	2.0 (1.0–3.0)	5.0 (2.0–5.0)	2.5 (1.8–3.5)
Straining	1.0 (0.0–2.0)	31/56 (55%)	1.0 (0.0–1.8)	3.5 (0.5–4.8)	0.5 (0.0–2.0)

Abbreviations: IQR, interquartile range; IPSS, International Prostate Symptom Score; MS, multiple sclerosis; RRMS, relapsing-remitting MS; SPMS, secondary progressive MS; PP, progressive MS (PPMS and PRMS combined).

Note: IPSS items: score 0–5; present if score > 0.

## DISCUSSION

The currents study is the first to evaluate both ED and LUTS in men with MS from Northeastern Brazil. The central finding is that neurological disability, as measured by the EDSS, emerged as the dominant determinant of urinary and sexual dysfunction, surpassing demographic factors such as age and disease duration. This observation has direct implications on how MS care integrates urological screening.

The 50% of ED prevalence in our cohort is consistent with the 49% pooled estimate reported in a meta-analysis of 2,760 men, 8 and it falls within the 31 at 92% range documented across 29 studies internationally. 17 This convergence across populations with diverse ethnic, geographic, and healthcare profiles suggests that ED in MS is primarily driven by a shared neurobiological substrate rather than by culturally-mediated or access-related factors. The higher prevalence reported by Tomé et al. 6 in Brazil (of 74.4%) likely reflects methodological differences, specifically the use of the full IIEF-15 with a more inclusive cut-off (≤ 45).

In the present study, the prevalence of LUTS (96.4%) is among the highest reported in the literature, in which rates typically range from 87% to 92% across studies employing validated instruments in diverse populations. 4 5 18 The near-universal prevalence in our cohort, with only 3.6% of patients symptom-free, exposes a fundamental gap between the actual burden of urinary dysfunction and what is captured through spontaneous patient reporting. This discrepancy underscores the inadequacy of relying on patients to volunteer urinary complaints, particularly given the well-documented reluctance to discuss such symptoms in MS consultations. 19

The strong EDSS–IIEF-5 correlation observed here is more robust than in most prior reports and supports a neuroanatomical model in which ED in MS results primarily from progressive disruption of spinal and supraspinal autonomic pathways. Adamec et al. 20 demonstrated that each EDSS point increase raised ED likelihood by 45.5%, and that preservation of the cremasteric reflex decreased ED risk by 61.9%, suggesting that the relationship between disability and ED reflects a dose-dependent loss of neural circuitry rather than a mere association. The parallel EDSS–IPSS correlation reinforces a similar disability-dependent trajectory for urinary dysfunction. 4 21 22 23

The IIEF-5–IPSS correlation is central to the clinical message of the current study: rather than representing independent complications, ED and LUTS appear to be interrelated manifestations of a shared pathophysiological process. The observed correlation establishes a co-variation between the two outcomes, while the inference of a common sacral autonomic substrate is supported by external urodynamic evidence rather than by direct measurement in our cohort; confirmation of this mechanistic interpretation in our population would require urodynamic and neurophysiological assessments, which were beyond the scope of the present study. Urodynamic evidence supports this interpretation, as involuntary detrusor pressure and reduced cystometric capacity independently predict moderate-to-severe ED, 24 and the IPSS independently predicts ED even after controlling for depression and EDSS. 25 Pilot data from a randomized trial 22 of tibial-nerve stimulation demonstrated concurrent improvement in urinary and sexual outcomes, suggesting that interventions targeting shared sacral circuitry may offer dual-benefit approaches worth pursuing in the Brazilian MS population.

The non-significant correlation between disease duration and the IIEF-5, while seemingly counterintuitive, is consistent with meta-regression data 26 showing negligible temporal effects on ED prevalence. It indicates that disability accumulation, not chronological disease duration, drives sexual dysfunction. Two patients with identical disease duration may present vastly different EDSS trajectories depending on phenotype, relapse severity, and treatment response. Conversely, disease duration did correlate with the IPSS, suggesting that urinary pathways may be more susceptible to cumulative subclinical damage over time, independently of discrete disability milestones. Two alternative explanations for the non-significant IIEF-5–disease duration association must be considered. First, patients with longer disease duration and severe disability are more likely to have been excluded by our criteria, introducing a survivor bias that may attenuate the apparent effect of time. Second, the influence of disease-modifying therapies and symptomatic treatments (which our database did not systematically capture) may have modified the natural trajectory of urogenital dysfunction in patients with longer disease duration.

The EDSS-stratified analysis reveals a non-linear, threshold-dependent relationship between disability and urogenital dysfunction: ED prevalence quadrupled, from 17.9% (95%CI: 7.9–35.6%) to 75% (95%CI: 50.5–89.8%) at the EDSS 3.0 boundary, and LUTS severity shifted from predominantly mild to universally-severe with EDSS ≥ 6.0. The near-complete overlap between the two dysfunctions provides direct clinical evidence that erectile dysfunction in MS seldom occurs as an autonomous phenomenon but rather as one component of a broader sacral autonomic failure syndrome. Our data suggest that an EDSS ≥ 3.0, instead of the traditional threshold ≥ 4.5 proposed by Fragalà et al., 24 may be a more sensitive clinical trigger for proactive dual screening, and the items on erection confidence and intermittency appear to capture the largest proportion of the variance related to disability in their respective instruments. These threshold estimates derive from a stratified subgroup, and the relatively-wide confidence intervals reflect this; replication in larger Brazilian cohorts is needed.

A practical implication of our findings is that validated screening instruments can be rapidly integrated into routine neurological consultations. The IIEF-5 and IPSS require minimal time to administer and provide clinically-actionable information. The correlation strengths observed suggest that any male patient with EDSS ≥ 3.0 should be proactively screened, regardless of spontaneous symptom reporting. The CREMPB has adopted this systematic approach since 2022, dedicating 8 minutes to each patient. This practice aligns with the Guidelines for Practising Neurologists on the Assessment and Treatment of Neurogenic Urinary and Sexual Symptoms (NEUROGED Guidelines) from the European Academy of Neurology 27 in 2025, which formally recommend systematic screening of neurogenic urogenital dysfunction in MS.

The item-level patterns provide preliminary hypotheses for differential therapeutic targeting. The low mean score in erection confidence suggests a psychogenic or anticipatory component potentially responsive to cognitive-behavioral or confidence-enhancing interventions in addition to phosphodiesterase type-5 inhibitor therapy. Conversely, the high severity of nocturia within the IPSS suggests that desmopressin or evening fluid-restriction protocols could be prioritized over generic anticholinergic regimens in this population. The shared sacral circuitry hypothesized to underlie ED and LUTS provides a rationale for dual-target interventions: the pilot randomized trial by Giannopapas et al. 22 demonstrated concurrent improvement in urinary and sexual outcomes with posterior tibial nerve stimulation, and our co-occurrence data support exploring such approaches in the Brazilian MS population. These therapeutic inferences should be regarded as hypothesis-generating and require validation in dedicated interventional studies.

The current study has limitations inherent to its cross-sectional design. The sample, while representing a substantial proportion of male MS patients in Paraíba, limited the multivariate regression power and resulted in small subgroup sizes for progressive phenotypes, precluding subgroup-specific multivariable modeling and rendering phenotype-specific conclusions tentative. Depression, an established independent predictor of ED, was not assessed with a validated instrument, representing a confounder that may have inflated the observed EDSS–urogenital correlations. Fatigue, another MS hallmark that may modulate sexual response, was similarly not measured, and urodynamic evaluation was not performed. The absence of a control group limits the attribution of dysfunction exclusively to MS; a comparison with published meta-analytic norms partially addresses this gap but remains indirect. Information on disease-modifying therapies and symptomatic treatments was not systematically captured; treatment effects may have modified the observed symptom severity in this cross-sectional snapshot. Additionally, all of our findings are specific to men with MS. The generalization to women, who present distinct pelvic neuroanatomical pathways, requires a sex-specific investigation.

Future studies should incorporate urodynamic evaluation, and validated depression and fatigue instruments. Longitudinal designs are needed to establish whether disability precedes urogenital dysfunction or whether both phenomena progress in parallel. Investigation of sacral neuromodulation and pelvic-floor rehabilitation in the Brazilian MS population is warranted. Multicenter Brazilian studies integrating cohorts from different regions would enable the assessment of ethnic, socioeconomic, and geographic influences on outcomes.

In conclusion, ED and LUTS are highly prevalent in men with MS in Paraíba, Brazil. The EDSS emerged as the strongest correlate of both dysfunctions, with a robust LUTS–ED nexus supporting the shared neural substrate hypothesis. These findings advocate for routine integration of brief screening instruments into neurological consultations, particularly for patients with progressive phenotypes and EDSS score ≥ 3.0.
